# Early introduction of food to prevent food allergy. The LEAP study (Learning Early about Peanut)

**DOI:** 10.1016/j.rpped.2015.07.002

**Published:** 2015

**Authors:** Nelson Rosário

**Affiliations:** a Universidade Federal do Paraná (UFPR), Curitiba, PR, Brazil. E-mail: nelson.rosario@onda.com.br

*Dear Editor*,

Peanut allergy is an increasing global health problem, which affects 1-3% of children in Western countries. The prevalence may have tripled in the last 15 years, which constitutes approximately 100,000 new cases annually in the USA and Canada.^1^^-^ 3

The most recent evidence, challenging the current guidelines, highlights the benefits of early introduction of peanuts, rather than avoidance, during the infants’ complementary food period. The basis is the study "Randomized Trial of Peanut Consumption in Infants at Risk for Peanut Allergy (Learning Early About Peanut - LEAP Trial)," which demonstrated an absolute decrease of 11-25% in the risk of developing peanut allergy in high-risk children if the food were introduced between 4 and 11 months of age.^4^ In the LEAP trial, 640 British infants at high risk for allergy, aged 4-11 months, were randomly assigned to consume products that contained peanut at least three times a week (6 g of peanut protein, the equivalent to 24 peanuts or 6 teaspoons of peanut butter a week) or to completely avoid products containing peanuts in the first five years of life. This population included 542 infants with negative skin prick tests at baseline and 98 infants with positive tests, with papule diameter of 1-4 mm to peanut extract. A total of 76 children whose skin tests showed papules larger than 4 mm in diameter, which implied a high probability of responding to peanut challenge, were excluded. In an intent-to-treat analysis, 17.2% of the group that avoided peanut developed a positive peanut challenge test at the age of 5 years compared to 3.2% in the group that consumed the food. This corresponded to a 14% reduction in the absolute risk of reaction, i.e., the necessary number (NNT) of 7.1 treated individuals for one to benefit from the treatment, and an 80% reduction in the relative risk.

The LEAP trial included only infants with minimal risk or negative skin tests and, therefore, it did not focus on a strategy for those without the risk factors to develop peanut allergy. This study is level 1 evidence for the practice of early introduction of peanut, which is safe and effective in selected infants at risk of developing allergy. This is the first prospective, randomized study on food allergy intervention and included those with positive skin tests, but no clinical reaction, with 80% reduction in the risk of developing peanut allergy.

Infants with early-onset allergic diseases, such as atopic dermatitis or food allergy in the first 4-6 months of life, should be evaluated by an allergist to implement LEAP trial suggestions.^5^^-^ 7 The evaluation of these patients should consist of skin tests and/or peanut ingestion in the doctor's presence to establish which ones are clinically reactive to the food, before starting its introduction at home.

There have been no studies with this level of accuracy in low-risk populations investigating the benefit of early introduction of peanuts. Another challenge is to establish in populations in which other foods are often allergy-causing, for instance, cow's milk, whether early intervention may reduce the prevalence of allergy to milk proteins or even other foods. The guidelines that deal with the time of introduction of foods into the infant's diet will be reviewed based on this new evidence.^8^^,^ 9
