# Supplementary material for: Early introduction of food to prevent food allergy. The LEAP study (Learning Early about Peanut)
Source: Rev Paul Pediatr. 2015 Oct-Dec;33(4):493–4. doi: 10.1016/j.rpped.2015.07.002 (PMC4685572; doi:10.1016/j.rpped.2015.07.002)
Supplement: Translation

# Translation: Introdução precoce do alimento para prevenção de alergia alimentar. O estudo LEAP (Learning Early about Peanut)

**Authors:** Nelson Rosário

**Affiliations:** a Universidade Federal do Paraná (UFPR), Curitiba, PR, Brasil. E-mail: nelson.rosario@onda.com.br

*Caro Editor*,

Alergia ao amendoim é um problema global crescente de saúde que afeta 1-3% das crianças nos países ocidentais. A prevalência pode ter triplicado nos últimos 15 anos, o que se traduz em cerca de 100.000 novos casos anualmente nos EUA e no Canadá.^1^^-^ 3

A evidência mais recente, que desafia as diretrizes em vigor, destaca os benefícios da introdução precoce, e não a tardia, do amendoim durante o período de complementação alimentar do lactente. A base é o estudo *Randomized Trial of Peanut Consumption in Infants at Risk for Peanut Allergy (Learning Early About Peanut - LEAP Trial)*, que demonstrou a redução absoluta de 11-25% no risco de desenvolver alergia ao amendoim em crianças de alto risco, se o alimento fosse introduzido entre quatro e 11 meses de idade.^4^ No estudo LEAP, 640 lactentes ingleses de alto risco de alergia, entre quatro e 11 meses, foram randomizados para consumir produtos com amendoim pelo menos três vezes por semana (6g de proteína de amendoim, equivalente a 24 amendoins ou seis colheres de chá de pasta de amendoim por semana) ou para evitar completamente produtos contendo amendoim pelos primeiros cinco anos de vida. Essa população incluiu 542 lactentes com testes cutâneos por puntura negativos à entrada do estudo e 98 lactentes com testes positivos, com diâmetro de pápulas ao extrato de amendoim de 1-4mm. Foram excluídas 76 crianças cujos testes cutâneos tinham pápulas maiores do que 4 mm em diâmetro, o que presumia alta probabilidade de reagir à provocação com amendoim. Em análise de intenção ao tratamento, 17,2% do grupo que evitava amendoim comparados com 3,2% do grupo que consumia o alimento desenvolveram teste de provocação positivo ao amendoim aos cinco anos. Isso correspondeu à redução de 14% do risco absoluto de reação, ou seja, ao número necessário (NNT) de 7,1 tratados para um ser beneficiado pelo tratamento e pela redução do risco relativo de 80%.

O estudo LEAP incluiu somente lactentes com risco mínimo ou testes cutâneos negativos e, portanto, não focou estratégia para aqueles sem os fatores de risco para desenvolverem alergia ao amendoim. Este estudo tem nível de evidência 1 para a prática de introdução precoce do amendoim, que é segura e efetiva em lactentes selecionados com risco de desenvolver alergia. Trata-se do primeiro estudo prospectivo, randomizado, de intervenção à alergia alimentar e incluiu aqueles com testes cutâneos positivos, mas sem reação clínica, e houve redução de 80% do risco de desenvolverem alergia ao amendoim.

Lactentes com doença alérgica de início precoce, tais como dermatite atópica ou alergia alimentar nos primeiros 4-6 meses de vida, devem ser avaliados por alergista para implantar as sugestões do estudo LEAP.^5^^-^ 7 A avaliação desses pacientes deve consistir em aplicação de testes cutâneos e/ou ingestão do amendoim na presença do médico para estabelecer quem é clinicamente reativo ao alimento antes de iniciar sua introdução em domicílio.

Não há estudos com esse rigor em populações de baixo risco que investigue o benefício da introdução precoce de amendoim. Outro desafio é estabelecer em populações nas quais outros alimentos são mais frequentemente causadores de alergia, por exemplo, o leite de vaca, se a intervenção precoce pode reduzir a prevalência de alergia às proteínas do leite ou mesmo de outros alimentos. As diretrizes que tratam da época de introdução de alimentos na dieta do lactente serão revistas com base nessa evidência apresentada.^8^^,^ 9
